# Age at Adiposity Rebound Is Associated with Fat Mass in Young Adult Males—The GOOD Study

**DOI:** 10.1371/journal.pone.0049404

**Published:** 2012-11-14

**Authors:** Claes Ohlsson, Mattias Lorentzon, Ensio Norjavaara, Jenny M. Kindblom

**Affiliations:** 1 Center for Bone and Arthritis Research, Institute of Medicine, The Sahlgrenska Academy at Gothenburg University, Gothenburg, Sweden; 2 Gothenburg Paediatric Growth Research Center, Institute of Clinical Sciences, The Sahlgrenska Academy at Gothenburg University, Gothenburg, Sweden; University of Santiago de Compostela School of Medicine - CIMUS, Spain

## Abstract

**Objective:**

Age at adiposity rebound (AR) is associated with obesity and Type 2 Diabetes in adults. The aim of the present study was to investigate the role of age at AR in adult fat mass, fat distribution and pubertal timing for a Swedish cohort.

**Patients and Methods:**

This is a retrospective cohort study. Detailed growth charts were retrieved for the men participating in the population-based GOOD (Gothenburg Osteoporosis and Obesity Determinants) study (n = 573). Body composition was analysed using dual X-ray absorptiometry and computed tomography at 18–20 years of age. Age and BMI at AR were calculated using pediatric growth charts and AR was defined as the lowest BMI between 3 and 9 years of age.

**Results:**

Subjects were divided into early (age at AR below 5.4 years of age), middle (age at AR 5.4 to 6.8 years of age) and late (age at AR after 6.8 years of age) age at AR tertiles. Subjects in the early age at AR tertile had higher young adult BMI (+8%), whole body fat mass (+34%) and amount of subcutaneous adipose tissue (+61%) than the subjects in the middle and late tertiles (p<0.01). The early age at AR tertile had an increased risk of obesity (Odds Ratio 4.1 [95% CI 1.2–13.9]) compared with the middle and late tertiles. In addition, the early age at AR tertile had Peak Height Velocity (PHV) 7 months earlier than the late tertile.

**Conclusions:**

Early age at AR was associated with young adult obesity as a consequence of a high amount of subcutaneous adipose tissue in men. In addition we made the novel observation that early age at AR was associated with an early puberty in men.

## Introduction

Childhood obesity has developed into an epidemic in the Western world and it carries a substantial risk of tracking into adulthood [1], [2]. Therefore it is of importance to find tools for early identification of individuals at risk. Adiposity rebound (AR) is the nadir of the BMI (Body Mass Index) curve during childhood, and has been found to predict both adult BMI [3]–[6] and adult obesity [6]–[8]. BMI at AR is also associated with adult BMI [4], [5]. Some studies, therefore, investigated the independent role for age at AR adjusting for BMI at AR. In a cohort including both males and females it was demonstrated that age at AR was an independent predictor for adult obesity [8]. In contrast, in the Bogalusa Heart Study including 105 children followed longitudinally, age at AR was no longer a significant predictor of adult BMI after adjustment for BMI at AR [4], [8]. Thus, whether or not age at AR is an independent predictor of adult BMI and body composition remains unclear.

Since age at AR is associated with both adult BMI and obesity, its impact on body composition has been investigated using anthropometrical measurements. In a study including 458 longitudinally followed subjects from New Zealand, subjects with early age at AR had larger waist circumference at adult age [9] than subjects with middle or late age at AR, and in the Bogalusa Heart study age at AR was inversely associated with subscapular skinfold thickness [4]. However, the role for age at AR for measurements of adult body composition using Dual X-Ray Absorptiometry (DXA) or computed tomography (CT) has not been investigated [4], [9].

Importantly, age at AR has also been linked to increased risk of developing Type 2 Diabetes. In a cohort including subjects from Finland born between 1934–44, age at AR before 5 years of age was associated with an incidence of 8.6% to develop type 2 diabetes, as compared with an incidence of 1.8% when AR occurred after 7 years of age [3]. An increased risk of developing Type 2 Diabetes in subjects with early age at AR has been confirmed in a British birth cohort [10].

Moreover, girls with early age at AR have earlier menarche [6], but whether or not age at AR is associated with pubertal timing in boys is not known.

The purpose with the present study was to investigate the independent role of age at AR for body composition in young adulthood and pubertal timing, using the population-based *G*öteborg *O*steoporosis and *O*besity *D*eterminants Study (the GOOD Study) including 573 males aged 18–20.

## Materials and Methods

### Study Design, Setting and Study Population

In the present retrospective cohort study, the GOOD study, 1068 male subjects aged 18–20 were enrolled [11], [12]. Study subjects from the city of Gothenburg were randomly identified using national population registers, contacted by telephone and asked to participate in this study. Inclusion criteria were age 18–20, male sex and willing to participate. There were no exclusion criteria. 48.6% of study subject candidates were included. At inclusion, study subjects were thoroughly characterized including anthropometrics, questionnaires regarding physical activity and diet, and examined using DXA (Dual X-Ray Absorptiometry), pQCT (peripheral Quantitative Computer Tomography) and abdominal CT (Computed Tomography). Given the retrospective cohort design, the measurements at young adult age performed at study start represent the most recent measurements in the study. Archived pediatric growth charts from visits at Child Health Care (CHC) centers were then retrieved for the study subjects. The CHC centers in Sweden represent a well-established organization that offers a base program of vaccinations and regular health visits including measurements of weight and height until the child is 5–6 years old. As many as 99% of children aged 0–5 participate [13]. Specially trained nurses carries out measurements of height and weight.

The GOOD study was approved by the local ethics committee at Gothenburg University. Written and oral informed consent was obtained from all the study participants.

### Exposure and Confounder Variables

In the present study we wanted to determine the association between age at Adiposity Rebound (AR) and adult body composition. Age at AR was therefore the exposure variable and in order to test if age at AR independent of BMI at AR predicts adult body composition, we adjusted for the confounding variable BMI at AR.

### Outcome Variables

The associations between the exposure variable age at AR and the confounder variable BMI at Adiposity Rebound were tested on outcome variables. The outcome variables in the present study were peak height velocity (PHV) and variables related to body composition at young adult age such as Body Mass Index (BMI), measurements using DXA, pQCT and abdominal CT, and serum leptin levels.

### Anthropometrical Measurements

Young adult height was measured using a wall-mounted stadiometer and weight was measured to the nearest 0.1 kg as previously described [11], [12] and was used for calculation of young adult BMI.

### Dual X-Ray Absorptiometry (DXA)

Total body fat mass, percentage body fat, total body lean mass and fat mass of the trunk, leg and arm were assessed for all subjects using the Lunar Prodigy DXA (GE Lunar Corp., Madison, WI USA).

### Peripheral Computer Tomography (CT) Analyses of Cross-sectional Adipose Tissue Area in the Distal Arm and Leg

A peripheral CT device (XCT-2000, Stratec Medizintechnik, GmbH, Pforzheim, Germany) was used to scan the distal leg and the distal arm of the non-dominant leg and arm, respectively, as previously described [12].

### Abdominal CT Analyses of Cross-sectional Adipose Tissue Areas

A previously described CT technique was used to measure the cross-sectional adipose area of the abdomen [12], [14], [15]. First, subcutaneous and visceral adipose tissue areas were measured, and subsequently the visceral adipose tissue area was divided into an intraperitoneal and a retroperitoneal adipose tissue area.

### Leptin Analysis

Serum was obtained from whole blood using standard procedures, frozen without delay, and stored at −70°C. Leptin was analyzed in serum samples as previously described [16].

### Estimation of Childhood BMI, Age and BMI at AR and PHV

Pediatric growth charts with longitudinal measurements of height and weight were collected retrospectively for the subjects of the GOOD study. The included study subjects had on average 27 height measurements (range 11–41), and 27 weight measurements (range 15–41). The measurements were performed by nurses as part of the children’s regular health visits at the CHC centers. Height measurements were curve-fitted according to the Infancy- Childhood- Puberty (ICP) model [17] as previously described [12], [18]. Age at Peak Height Velocity (PHV) was defined as age at the maximum growth velocity during puberty and was used for assessment of pubertal timing.

Childhood body weight was computed through fitting of the weight curve for each child using smooth splines (smooth.spline in the R package statistics, the R foundation for statistic computing, Vienna, Austria; www.r-project.org). Childhood BMI was then calculated from the estimated values of weight and height. Age at AR was defined as age at the lowest BMI between 3 and 9 years of age and was then computed using the curve-fitting program.

### Statistical Analysis

The associations between the exposure variable (age at AR) and the outcome variables were first tested using Pearson’s correlation analyses. Linear regression analyses were then performed between the exposure variable and body composition variables. The model used was a simple predictive linear regression, either without ( = crude), or with, adjustment for BMI at Adiposity Rebound ( = adjusted). Adjustment for BMI at AR was performed in order to determine whether or not age at AR was independently associated with the outcome variables. Unstandardized β-values are given.

Body composition variables were age-adjusted according to young adult age at measurements (ie prior to statistical analysis in the present study). We tested for normal distribution. With the exception of height, lean mass and age at PHV, none of the dependent variables displayed normal distribution. Therefore the variables have been log-transformed.

Tertiles of age at AR, and the cohorts (entire GOOD cohort, AR cohort and CT cohort) were compared using one-way ANOVA followed by Tukey’s Post-Hoc test. The Z-score referred to is the Z-score of BMI in the study population. Odds ratios for overweight and obesity were tested using a logistic regression model.

All statistical calculations were performed using age-adjusted body composition variables. BMI and fat parameters, derived from body composition analyses, were not normally distributed and have therefore been log-transformed. Values are given as means ± SD and/or median and interquartile range (25^th^–75^th^ percentiles) unless otherwise stated. For all the statistical analyses the software SPSS (version 17.0) was used.

## Results

Age at AR was 6.1±1.3 years of age in the subsample (n = 573) of the GOOD Study with enough growth and weight data to calculate AR. The impact of age and BMI at AR, derived from retrospective childhood data, on adult BMI and fat parameters such as percentage body fat, fat mass trunk ( = central fat), fat mass arm and leg ( = peripheral fat), and abdominal adipose tissue depots ( = subcutaneous (ScAT) and visceral; ie intraperitoneal (IpAT) and retroperitoneal adipose tissue areas (RpAT) and with serum levels of leptin at young adult age were investigated.

To determine whether the GOOD cohort was representative of the general young male population in Gothenburg, we compared the height and weight of the GOOD subjects with a group of 624 age-matched, randomly selected conscripts (86% of all Swedish men underwent testing for military service), living in the same area as the GOOD subjects. The differences in height, weight or BMI were small and not clinically significant, showing that the GOOD cohort is representative of the general young male population of Gothenburg [11].

**Figure 1:**
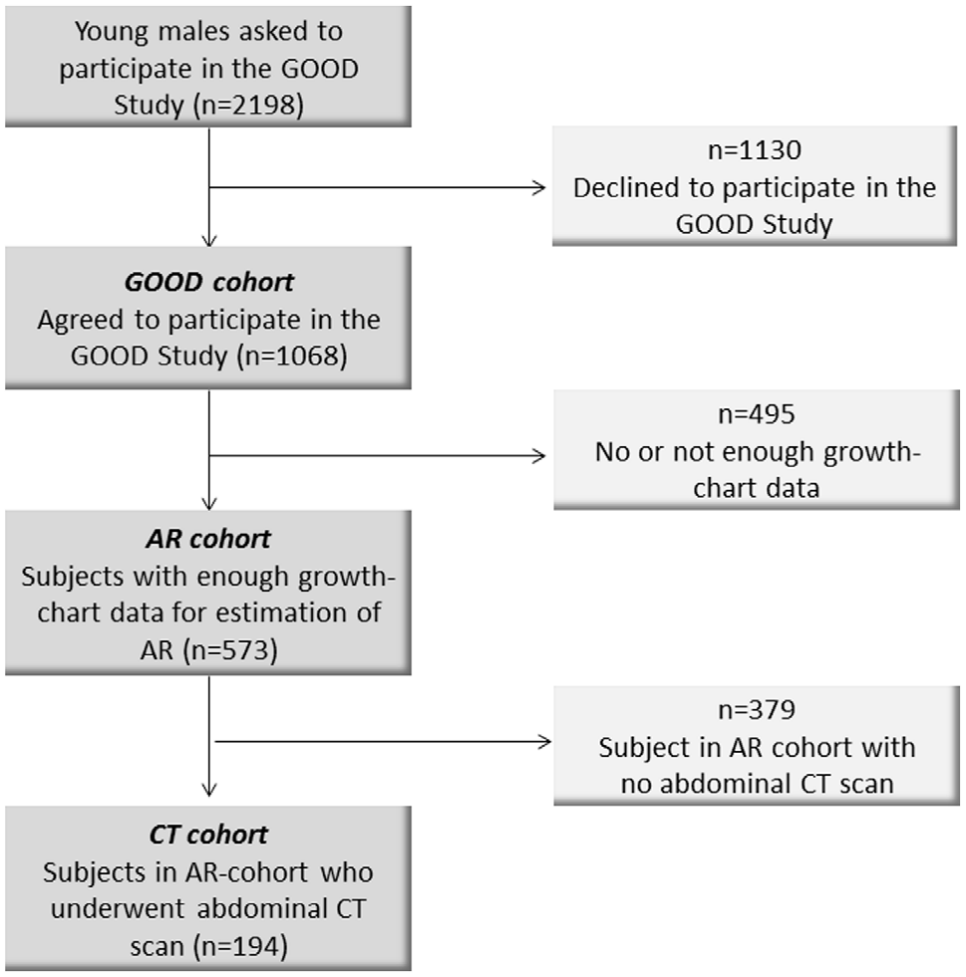
Flowchart of study. GOOD = Gothenburg Osteoporosis and Obesity Determinants Study, AR = Adiposity Rebound, CT = Computed Tomography.

Of the 1068 subjects ( = GOOD cohort), complete growth and weight charts for determination of adiposity rebound were available for 573 subjects ( = AR cohort). Thus, the results presented here were obtained from a subsample of the original GOOD cohort (Figure 1). Abdominal CT scans were performed on 194 ( = the CT cohort) of the 573 study subjects in the AR cohort (Figure 1). The differences between the AR cohort, the CT cohort and the entire GOOD cohort in terms of height, weight and BMI were small and were not considered as clinically significant. The CT cohort was slightly younger that the AR cohort and the entire GOOD (p<0.001) but was not considered clinically significant (Table S1). Thus, the AR cohort is considered representative of the GOOD cohort, and the CT cohort is representative of both the AR cohort and the GOOD cohort (Table S1).

General anthropometrics for the AR cohort are presented in Table 1.

**Table 1:** Anthropometrics and fat variables.

Variables	Median(IQR)
**Adiposity Rebound** (n = 573)	
Age at AR (years)	6.2 (5.1–7.0)
BMI at AR (kg/m^2^)	15.1 (14.4–15.9)
**Age at PHV** (years; n = 466)	13.6 (12.9–14.2)
**Serum leptin** (ng/ml, n = 573)	5.1 (3.3–7.79)
**Adult Anthropometrics** (n = 573)	
Age (years)	18.8 (18.4–19.3)
Height (cm)	181.2 (177.0–186.2)
Weight (kg)	71.2 (65.8–78.6)
Young adult BMI (kg/m^2^)	21.6 (20.1–23.6)
**Adult DXA** (n = 573)	
Whole body lean mass (kg)	56.9 (52.8–61.2)
Whole body fat mass (kg)	11.0 (7.8–15.2)
Percentage body fat (%)	15.5 (11.5–20.4)
Fat mass trunk (kg)	5.4 (3.8–7.7)
Fat mass arm (kg)	0.4 (0.3–0.6)
Fat mass leg (kg)	2.1 (1.5–2.9)
**Adult Peripheral CT** (n = 573)	
ScAT leg (cm^2^)	13 (10–16)
**Adult Abdominal CT** (n = 194)	
Total AT (cm^2^)	115 (77–166)
ScAT (cm^2^)	78 (51–125)
IpAT (cm^2^)	31 (23–44)
RpAT (cm^2^)	19 (14–27)

Values are given as median and Inter Quartile Range (IQR). BMI = body mass index, AR = adiposity rebound, PHV = Peak Height Velocity, DXA = Dual X-Ray Absorptiometry, CT = computed tomography. Sc = subcutaneous, Ip = intraperitoneal, Rp = retroperitoneal, AT = adipose tissue.

### Early Adiposity Rebound is Associated with High Fat Mass

#### Correlation analyses

Age at AR was inversely associated with young adult BMI and explained 7.8% of the variance in young adult BMI (Table 2). Age at AR derived from retrospective childhood data was also associated with body composition. It predicted young adult whole body fat mass, percentage body fat and both trunk and peripheral (leg and arm) fat mass (Table 2). We then investigated if age at AR was associated with young adult adipose tissue depots as measured with abdominal CT. These analyses revealed that age at AR predicted the total adipose tissue area and the Subcutaneous Adipose Tissue area (ScAT) at young adult age. The variance in ScAT explained by age at AR was 8.0%. In contrast, parameters reflecting visceral fat (IpAT and RpAT) were not significantly associated with age at AR (Table 2). Correlation analyses between serum leptin levels and body composition variables are shown in Table S2.

**Table 2:** Correlation analyses for age at AR.

Variables	Pearson’s r	p-value
**Adult BMI** (kg/m^2^)	−0.28	<0.001
**Adult DXA**		
Whole body fat mass (kg)	−0.25	<0.001
Percentage body fat (%)	−0.23	<0.001
Fat mass trunk (kg)	−0.25	<0.001
Fat mass arm (kg)	−0.23	<0.001
Fat mass leg (kg)	−0.23	<0.001
**Adult Peripheral CT**		
ScAT leg (cm^2^)	−0.14	0.001
**Adult Abdominal CT**		
Total AT (cm^2^)	−0.26	<0.001
ScAT (cm^2^)	−0.28	<0.001
IpAT (cm^2^)	−0.14	NS
RpAT (cm^2^)	−0.08	NS
**Age at PHV** (years)	0.24	<0.001

Pearson’s correlation coefficients are shown for associations with age at AR. All variables except age at AR and age at PHV have been log-transformed. AR = Adiposity rebound, BMI = body mass index, PHV = Peak Height Velocity, Sc = subcutaneous, Ip = Intraperitoneal, Rp = Retroperitoneal, AT = adipose tissue.

### Age at Adiposity Rebound Adjusted for BMI at AR Predicts Fat Mass

#### Linear regression model

To explore whether or not age at AR predicts young adult fat mass, we performed linear regression analyses. The linear regression analyses were performed both unadjusted ( = crude) and adjusted for BMI at AR. This adjustment was performed as age at AR was associated with young adult BMI at AR (*Pearson’s r* = −0.18, p<0.001). Our results demonstrated that age at AR is a strong, negative predictor of young adult BMI and the association was maintained after adjustment for BMI at AR (Table 3). Thus, age at AR, adjusted for BMI at AR, was a predictor of young adult BMI and for every year of earlier age at AR, young adult BMI was 0.63 kg/m^2^ higher. Similarly, age at AR was a negative predictor of variables of young adult body composition such as fat mass and percentage body fat. For every year of earlier age at AR, young adult fat mass was 1.3 kg higher. Addition of age at AR to regression models including BMI at AR, demonstrated that age at AR explained an additional 4.1% of the variance in young adult percentage body fat (p<0.001). Moreover, age at AR was a negative predictor of variables of central (trunk fat mass) as well as peripheral (leg and arm) fat mass both unadjusted and adjusted for BMI at AR (Table 3). When the role of age at AR was investigated for specific abdominal fat depots, we found that age at AR, adjusted for BMI at AR was a significant negative predictor of young adult ScAT but not of IpAT or RpAT (Table 3).

**Table 3:** Linear regression analyses.

Variables	Crude	Adjusted for BMIat AR
	β-value (95% CI)	β-value (95% CI)
**Adult BMI** (kg/m^2^)	−0.63 (−0.81; −0.45)	−0.44 (−0.61; −0.28)
**Adult DXA**		
Whole body fat mass (kg)	−1.3 (−1.8; −0.9)	−1.1 (−1.5; −0.7)
Percentage body fat (%)	−1.2 (−1.7; −0.8)	−1.1 (−1.5; −0.7)
Fat mass trunk (kg)	−0.74 (−0.97; −0.51)	−0.62 (−0.85; −0.39)
Fat mass arm (kg)	−0.06 (−0.08; −0.04)	−0.05 (−0.07; −0.03)
Fat mass leg (kg)	−0.23 (−0.31; −0.15)	−0.18 (−0.26; −0.10)
**Adult Peripheral CT**		
ScAT leg (cm^2^)	−0.54 (−0.82; −0.25)	−0.37 (−0.65; −0.09)
**Adult Abdominal CT**		
Total AT (cm^2^)	−18 (−29; −8)	−14 (−25; −4)
ScAT (cm^2^)	−17 (−25; −8)	−13 (−22; −4)
IpAT (cm^2^)	−1.2 (−2.5; 0.1)	−0.9 (−2.2; 0.5)
RpAT (cm^2^)	−0.5 (−1.4; 0.4)	−0.4 (−1.3; 0.6)
**Age at PHV** (year)	0.19 (0.12; 0.26)	0.18 (0.11; 0.25)

Linear regression including only age at AR (adiposity rebound; = Crude) and after adjustment for body mass index (BMI) at adiposity rebound ( = adjusted for BMI at AR). Values are given as unstandardized β-values expressed per year. BMI = body mass index, CI = confidence interval, DXA = Dual X-ray Absorptiometry, CT = Computed Tomography, Sc = subcutaneous, Ip = intraperitoneal. Rp = retroperitoneal, AT = adipose tissue, PHV = Peak Height Velocity.

### Different Childhood BMI Trajectories for Tertiles of Age at AR

**Figure 2:**
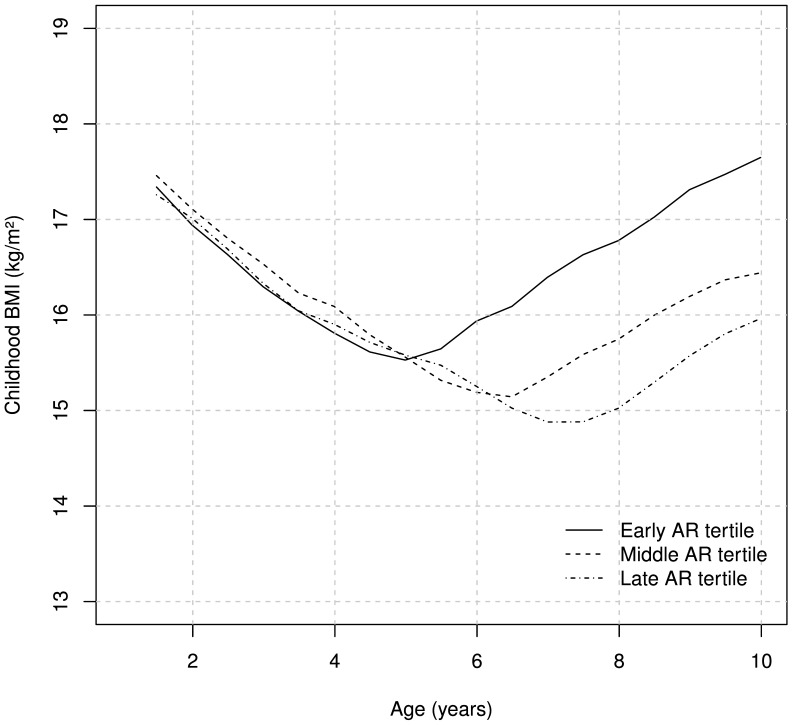
Childhood BMI plotted for tertiles of age at AR. Values are given as means. BMI = Body Mass Index, AR = Adiposity rebound. p<0.001 from 6 years of age for the middle and late AR tertiles versus the early AR tertile. p<0.05 from 7 years of age for the late AR tertile versus the middle, and for middle AR tertile versus early at 4 years of age.

**Figure 3:**
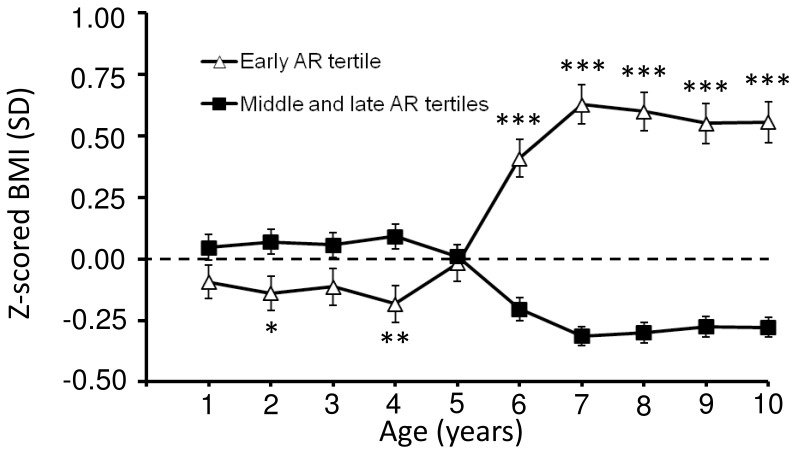
Standardized childhood BMI for early age at AR versus middle and late age at AR. Values are given as Z-score (SD) means ± SEM. BMI = Body Mass Index, AR = Adiposity rebound. *p<0.05, **p<0.01 and ***p<0.001 vs. middle and late tertiles.

When subjects were divided into tertiles according to age at AR and childhood BMI was plotted for each tertile, BMI during childhood displayed a different trajectory depending on early, middle or late AR (Figure 2). The early AR tertile (age at AR before 5.4 years of age) had the nadir at an average BMI of 15.4±1.1 kg/m^2^. The middle AR tertile (age at AR between 5.4 and 6.8 years of age) had an average BMI of 15.1±1.0 kg/m^2^ (p = 0.006 against BMI in the early age at AR tertile), and the late AR tertile (age at AR after 6.8 years of age) had a BMI of 14.9±1.1 kg/m^2^ (p<0.001 against early age at AR tertile; Figure 2). After six years of age, subjects in the early age at AR tertiles had significantly higher BMI than subjects both in the middle and late age at AR tertiles (Figure 2). Interestingly, subjects in the early age at AR tertile had slightly lower childhood BMI than subjects in the middle age at AR tertile at four years of age (p<0.05). In order to further characterize the role of early age at AR for childhood BMI, standardized BMI (Z-scores) were plotted for both the early age at AR tertile and the middle and late age at AR tertiles together, demonstrating that subjects with early age at AR had a higher BMI from six years of age but a lower BMI during early childhood (2 and 4 years of age) than subjects with middle or late age at AR (Figure 3).

### Tertiles of Age at AR and Young Adult Fat Characteristics

**Figure 4:**
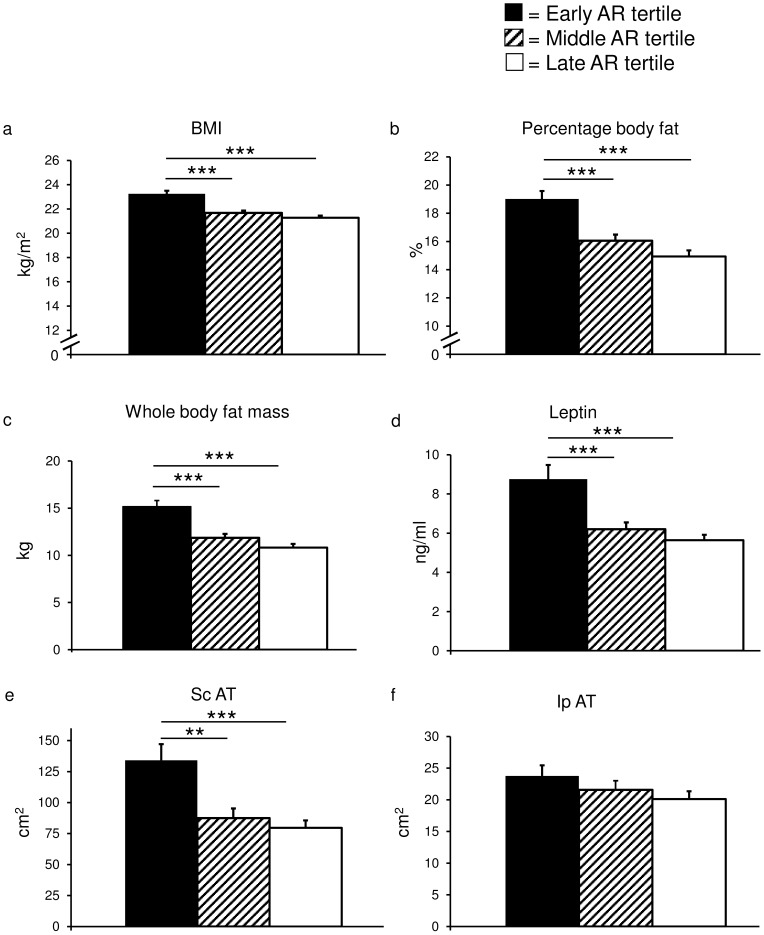
Fat parameters according to age at AR tertiles. Histograms representing early, middle and late age at adiposity rebound tertiles for BMI (a), percentage body fat (b), whole body fat mass (c), serum leptin levels (d), Subcutaneous adipose tissue (ScAT; n = 194; e) and Intraperitoneal adipose tissue (IpAT; n = 194; f). Values are given as means ± SEM. BMI = Body Mass Index, **p<0.01, ***p<0.001.

To illustrate the differences in fat characteristics between subjects with early, middle and late age at AR, subjects were evaluated according to tertiles of age at AR demonstrating that the early age at AR tertile had a higher BMI (+8%), percentage body fat (+23%) and whole body fat mass (+34%) than the middle and late age at AR tertiles (Figure 4a, b, and c). Serum levels of leptin, an adipocyte-derived hormone, showed the same pattern as BMI and fat mass and were higher in the early age at AR tertile than in the middle and late age at AR groups (Figure 4d). The differences in fat mass and percentage body fat between tertiles of age at AR were reflected by 61% higher ScAT in the early age at AR group compared with the middle and late age at AR tertiles (Figure 4e) but no significant differences between tertiles were seen for IpAT (Figure 4 f) or RpAT (data not shown). No significant difference in BMI, fat mass, percentage body fat, leptin or ScAT was seen between the middle and late age at AR tertiles (Figure 4).

### Early AR is Associated with Obesity

In logistic regression models, the odds ratios for overweight (BMI>25 kg/m^2^) and obesity (BMI>30 kg/m^2^) in the early age at AR tertile compared with the middle and late tertiles were 3.2 (95% CI 1.9–5.2) and 4.1 (95% CI 1.2–13.9), respectively. Thus, early age at AR is associated with an increased risk of obesity.

### Early AR is Associated with Early Pubertal Timing

Finally, we evaluated if age at AR predicted pubertal timing. Age at AR was directly associated with age at peak height velocity (PHV; Table 2) and could explain as much as 5.9% (p<0.001) of the variance in age at PHV. In the linear regression model age at AR was a predictor of age at PHV independent of BMI at AR (Table 3). For the early tertile, age at PHV was at 13.3±1.0 years, for the middle tertile at 13.6±1.1 years and for the late tertile at 13.9±0.9 years (p = 0.018 early versus middle, p<0.001 early versus late and p = 0.08 middle versus late). The early age at AR tertile had PHV 7 months earlier than the late tertile.

## Discussion

Our results indicate that age at AR is a negative predictor of not only young adult BMI but also of whole body fat mass, trunk fat mass, peripheral fat mass and serum leptin levels in a large population-based cohort of young adult Swedish males independent of BMI at AR. Using abdominal CT analyses we have studied the role of age at AR for the subcutaneous and visceral adipose tissue separately. Interestingly, early AR is associated with subcutaneous but not visceral fat mass in young adult Swedish males. In addition we made the novel observation that early age at AR is associated with an early puberty in men.

A multitude of studies have confirmed the role for age at AR in predicting adult BMI, obesity and Type 2 Diabetes but the association between timing of AR and adult fat mass is not thoroughly studied. Previous studies have indicated an association between waist circumference at adult age and age at AR [9] and an inverse association between age at AR and subscapular skinfold thickness in adult age [4], indicating an association between timing of AR and indirect measurements of fat mass. We have performed direct measurements of fat mass using DXA and our results indicate that subjects with early age at AR have higher fat mass. For every year of earlier age at AR, fat mass was increased by 1.3 kg. Indicators of fat mass derived from anthropometric measurements, such as skinfold thickness and waist circumference, cannot separate subcutaneous and visceral adipose tissue. In the present study we therefore measured the individual depots of adipose tissue using abdominal CT and demonstrate an inverse association between timing of AR and subcutaneous fat. Subjects with early age at AR have 61% larger ScAT than those with middle or late age at AR. Interestingly, there was no significant association between timing of AR and visceral fat mass. While visceral fat is a strong risk factor for cardiovascular disease and the metabolic syndrome, it has recently been shown that expanded subcutaneous adipose tissue as well is associated with increased cardiovascular risk [19]. The results from the present study indicate that early age at AR predicts overweight and obesity. We believe that identification of individuals with an early age at AR, for example age at AR before 5 years of age, might be useful and together with other measures contribute to the prevention of overweight/obesity and eventually also cardiovascular and metabolic risks.

Previous studies of the role for age at AR have demonstrated an association with adult obesity and risk of adult Type 2 Diabetes. However, while one study demonstrated an independent role of age at AR for adult obesity [8] and one study failed to demonstrate an independent role of age at AR after adjusting for BMI at AR [4], most studies have not investigated the role of age at AR, independent of BMI at AR, on adult fat mass. Concerns have therefore been raised about the lack of evidence linking an early age at AR to adult fat mass [20] and some critics have argued that age at AR is an epiphenomenon only seen using population statistics with little use in individuals [20]. Others mean that an early age at AR predicts BMI and obesity because “it identifies children whose BMI centile is high and/or crossing upwards” [21]. In the present study we found that age at AR is a negative predictor of both adult BMI, adult fat mass and adult ScAT, independent of BMI at AR. Our results also indicated that age at AR could explain an additional 4.1% of the variance in young adult percentage body fat. Thus, timing of AR predicts young adult fat mass and contributes unique information not given by BMI at AR.

A link between age at menarche and body fat was proposed already in the 70s, and is thought to be mediated through leptin [22], but the role of fat mass for the initiation of puberty in boys is unclear [23]. In the present study we show, for the first time, an association between early age at AR and an early puberty in men. The difference between the early and late age at AR tertiles was 7 months in pubertal timing. Since the present study is retrospective with respect to childhood data, fat mass during adolescence has not been measured. However, we can link an early AR with a subsequent rise in BMI in the time period between age at AR and pubertal onset. In addition, our findings indicate that subjects with early age at AR have the highest amounts of body fat in young adult age. Our findings lend support to the notion that body fat might be of importance for the initiation of puberty in boys as well as in girls.

In the GOOD study, the early age at AR tertile had the highest young adult BMI, but also had slightly lower BMI before 4 years of age. One may speculate that there is an intrinsic programming for the trajectory of childhood BMI, but which factors that are involved in regulating childhood BMI and timing of AR is not known.

The main limitation with the present study is that findings from association studies cannot establish any causal relationships and interpretations should therefore be made with caution. Another limitation is that data was collected retrospectively from pediatric growth charts, and that therefore data on some potential confounding factors during childhood, such as diet and exercise, is lacking. The possibility of bias in number of measurements related to the presence or absence of obesity could not be ruled out. However, the findings from this study add new knowledge on how age at AR associates with young adult body composition and fat characterization and with overweight/obesity. We believe that detection of an early age at AR might be one of the tools for the clinician to identify a non-beneficial weight development during childhood. More studies will be necessary to investigate the effect of interventions and whether or not it is possible to shift age at AR to a later age by altering nutritional or environmental factors. The main strengths with the present study are the detailed growth charts, enabling calculations of age at AR and PHV, and the thorough fat phenotyping performed, including DXA and both peripheral and abdominal CT measurements.

In conclusion, early adiposity rebound is associated with young adult obesity as a consequence of a high amount of subcutaneous adipose tissue in men. In addition we made the novel observation that early age at AR is associated with an early puberty in men. These findings suggest that age at AR might be useful to identify boys at risk of future obesity.

## Supporting Information

Table S1 **Comparison of age, height, weight and BMI between cohorts and subsamples.** Comparison between the entire GOOD cohort (n = 1068), the sub-set in the present study (the AR cohort, n = 573) and the sub-set of the AR cohort who underwent abdominal CT-scans (CT cohort, n = 194). A One-Way ANOVA was followed by Tukey’s Post-Hoc test. *** p<0.001 versus the GOOD cohort (n = 1068), ### p<0.001 versus the AR cohort (n = 573). SD = Standard Deviation, IQR = Inter Quartile Range, AR = Adiposity Rebound, CT = Computer Tomography, BMI = Body Mass Index, NS = not significant.
(DOCX)

Table S2 **Correlation analyses between serum leptin levels and measurements of fat mass.** Pearson’s correlation coefficients are shown for associations between serum leptin levels and measurements of body fat. All variables have been log-transformed. AR = Adiposity rebound, BMI = body mass index, Sc = subcutaneous, Ip = Intraperitoneal, Rp = Retroperitoneal, AT = adipose tissue. p>0.001 for all correlations.
(DOCX)
