# Supplementary material for: Age at Adiposity Rebound Is Associated with Fat Mass in Young Adult Males—The GOOD Study
Source: PLoS One. 2012 Nov 14;7(11):e49404. doi: 10.1371/journal.pone.0049404 (PMC3498114; doi:10.1371/journal.pone.0049404)
Supplement: Table S1 — Comparison of age, height, weight and BMI between cohorts and subsamples. Comparison between the entire GOOD cohort (n = 1068), the sub-set in the present study (the AR cohort, n = 573) and the sub-set of the AR cohort who underwent abdominal CT-scans (CT cohort, n = 194). A One-Way ANOVA was followed by Tukey’s Post-Hoc test. *** p<0.001 versus the GOOD cohort (n = 1068), ### p<0.001 versus the AR cohort (n = 573). SD = Standard Deviation, IQR = Inter Quartile Range, AR = Adiposity Rebound, CT = Computer Tomography, BMI = Body Mass Index, NS = not significant. (DOCX) [file pone.0049404.s001.docx]

**S1 Comparison of age, height, weight and BMI between cohorts and subsamples**

| **Variable** | **GOOD**  **(n=1068)** | | **AR cohort**  **(n=573)** | | **CT cohort**  **(n=194)** | |
| --- | --- | --- | --- | --- | --- | --- |
|  | ***Mean (SD)*** | ***Median (IQR)*** | ***Mean (SD)*** | ***Median (IQR)*** | ***Mean (SD)*** | ***Median (IQR)*** |
|  |  |  |  |  |  |  |
| Age (years) | 18.9 (0.6) | 18.8 (18.4-19.3) | 18.9 (0.5) | 18.3 (18.4-19.3) | 18.7 (0.5)***### | 18.6 (18.3-19.0) |
| Height (cm) | 181.4 (6.8) | 181.6 (176.8-186.0) | 181.5 (6.7) | 181.2 (177.0-186.2) | 181.6 (6.8) | 181.8 (177.3-186.8) |
| Weight (kg) | 73.8 (11.9) | 71.9 (66.0-79.6) | 72.8 (11.0) | 71.2 (65.8-78.9) | 72.6 (11.3) | 70.8 (65.1-78.3) |
| BMI (kg/m^2^) | 22.4 (3.2) | 21.9 (20.3-23.9) | 22.1 (3.0) | 21.6 (20.1-23.6) | 22.0 (3.0) | 21.5 (20.0-23.6) |

Comparison between the entire GOOD cohort (n=1068), the sub-set in the present study (the AR cohort, n=573) and the sub-set of the AR cohort who underwent abdominal CT-scans (CT cohort, n=194). A One-Way ANOVA was followed by Tukey´s Post-Hoc test. *** p<0.001 versus the GOOD cohort (n=1068), ### p<0.001 versus the AR cohort (n=573).

SD= Standard Deviation, IQR= Inter Quartile Range, AR= Adiposity Rebound, CT=Computer Tomography, BMI= Body Mass Index, NS= not significant.
