# Supplementary material for: Age at Adiposity Rebound Is Associated with Fat Mass in Young Adult Males—The GOOD Study
Source: PLoS One. 2012 Nov 14;7(11):e49404. doi: 10.1371/journal.pone.0049404 (PMC3498114; doi:10.1371/journal.pone.0049404)
Supplement: Table S2 — Correlation analyses between serum leptin levels and measurements of fat mass. Pearson’s correlation coefficients are shown for associations between serum leptin levels and measurements of body fat. All variables have been log-transformed. AR = Adiposity rebound, BMI = body mass index, Sc = subcutaneous, Ip = Intraperitoneal, Rp = Retroperitoneal, AT = adipose tissue. p>0.001 for all correlations. (DOCX) [file pone.0049404.s002.docx]

**S2 Correlation analyses between serum leptin levels and measurements of fat mass**

|  |  | **Adult DXA** | | | | | | **Adult Abdominal CT** | | | | | | | |  |
| --- | --- | --- | --- | --- | --- | --- | --- | --- | --- | --- | --- | --- | --- | --- | --- | --- |
|  | **BMI** | **Fat mass** | | **Percentage body fat** | | **Fat mass trunk** | | **Total AT** | | **ScAT** | | **IpAT** | | **RpAT** | |  |
| **Leptin** | 0.48 | 0.62 | | 0.61 | | 0.60 | | 0.63 | | 0.62 | | 0.44 | | 0.49 | |  |
| **BMI** |  | 0.78 | | 0.66 | | 0.78 | | 0.76 | | 0.77 | | 0.50 | | 0.50 | |  |
| **Adult DXA** |  |  | |  | |  | |  | |  | |  | |  | |  |
| **Fat mass** |  |  | | 0.97 | | 0.99 | | 0.93 | | 0.94 | | 0.67 | | 0.64 | |  |
| **Percentage body fat** |  |  | |  | | 0.96 | | 0.92 | | 0.93 | | 0.67 | | 0.64 | |  |
| **Fat mass trunk** |  |  | |  | |  | | 0.94 | | 0.94 | | 0.69 | | 0.67 | |  |
| **Adult abdominal CT** |  |  | |  | |  | |  | |  | |  | |  | |  |
| **Total AT** |  |  | |  | |  | |  | | 0.98 | | 0.76 | | 0.76 | |  |
| **ScAT** |  |  | |  | |  | |  | |  | | 0.66 | | 0.68 | |  |
| **IpAT** |  |  | |  | |  | |  | |  | |  | | 0.59 | |  |
| **RpAT** |  |  | |  | |  | |  | |  | |  | |  | |  |
|  |  |  |  | |  | |  | |  | |  | |  | |  | |

Pearson´s correlation coefficients are shown for associations between serum leptin levels and measurements of body fat. All variables have been log-transformed. AR= Adiposity rebound, BMI=body mass index, Sc= subcutaneous, Ip=Intraperitoneal, Rp= Retroperitoneal, AT= adipose tissue. p>0.001 for all correlations.
